# A Reconfigurable Low-Pass Filter Based on Polyurethane Substrate Inspired by the Origami Structure

**DOI:** 10.3390/mi16091060

**Published:** 2025-09-18

**Authors:** Kang Wang, Mingcheng Li, Chuyuan Gao, Yupeng Dong, Yutang Pan, Ming Qin, Meng Nie, Lei Han

**Affiliations:** 1 Key Laboratory of MEMS of the Ministry of Education, Southeast University, Nanjing 210096, China; epiphanyer@163.com (K.W.); 220246483@seu.edu.cn (M.L.); 230238403@seu.edu.cn (C.G.); 13637091865@163.com (Y.D.); 220221727@seu.edu.cn (Y.P.); mqin@seu.edu.cn (M.Q.); 2 National Engineering Research Center of Communication Software and Asic Design, The 54th Research Institute of China Electronics Technology Group Corporation, Shijiazhuang 050081, China

**Keywords:** origami structure, reconfigurable, low-pass filter, V-shaped beam, rhombic beam

## Abstract

In this paper, an innovative reconfigurable microstrip RF device design method is proposed, which is inspired by origami structures. The experimental results of the reconfigurable low-pass filter indicate that the maximum origami folding height is 3 mm, resulting in the frequency tuning range of the filter being 524~568 MHz, the return loss is below −15.0 dB and the insertion loss is below 2.5 dB up to 500 MHz. It is demonstrated that the proposed design method for reconfigurable microstrip RF devices is fairly effective through theoretical and experimental research. This work provides a groundbreaking method for reconfigurable RF devices with origami structures.

## 1. Introduction

Reconfigurable filters play a pivotal role in wireless communication systems and microwave sensors [1], because of the increasingly rigorous demands of communication systems for frequency tuning [2] and self-adaption [3]. To achieve reconfiguration in microstrip filters, variable capacitors [4], variable inductors [5] and PIN diodes [6] are employed, which leads to high insertion loss due to complex device fabrication and parasitic effects. Therefore, the mechanical structure [7,8,9] is applied to realize physical isolation. However, traditional mechanical structures cannot achieve large displacement when driven in the vertical direction, which limits the improvement of RF devices microwave performance. In contrast, origami [10,11,12] can transform from a two-dimensional plane structure to a three-dimensional structure, effectively expanding the spatial advantage. Origami has been extensively researched, and the applications have been expanded to unexplored areas, such as optical equipment [12], space solar panels [13] and metamaterials [14]. Meanwhile, applications of origami structures have been investigated, such as self-folding [15,16,17] structures as substrates for microwave devices. However, these studies only apply origami structures to deformation, none of them have adopted origami for reconfigurable RF devices.

In this paper, an innovative reconfigurable microstrip RF device design method inspired by origami is introduced. The reconfigurable microstrip low-pass filter with an origami structure based on the design method is fabricated by laser-cutting, and the circuit structure is patterned by silk-screen printing. The experimental results agree with the simulated results, which demonstrates the validity of the proposed design method for reconfigurable microstrip RF devices.

## 2. Model and Design

### 2.1. Theoretical Model

The reconfigurable low-pass filter is composed of three parts as a V-shaped beam, a rhombic beam and an origami structure. The V-shaped beam is a driving structure used to generate initial displacement. When the current is applied to the anchors of the V-shaped beam, the current is converted into Joule heat, causing the thermal expansion of the V-shaped beam to generate a small output displacement. The V-shaped beam has a substantially greater length than its cross-sectional dimensions. Therefore, an electro-thermal analysis model is established using one-dimensional differential element theory. According to the computation of thermal expansion in the V-shaped beam, the displacement of the V-shaped beam along the direction of motion is calculated by

(1) $$D=\frac{L_{v}^{2}}{8EI}\left(\frac{L_{v}}{6}X^{2}-X^{3}\right)cos\theta$$

where $L_{v}$ is the length of the V-shaped beam, E is Young’s Modulus, X is the constraint force acting on the V-shaped beam, θ is the horizontal tilt angle of the beam with current and I is the second moment of area of the V-shaped beam.

The rhombic beam is introduced as a bridge between the V-shaped beam and the origami structure to amplify the displacement of the V-shaped beam to realize the ideal deformation of the origami structure. To establish the relationship among the displacement amplification ratio, the length and the included angle of the rhombic beam, the idealized model assumes purely rotational joints without internal stress dissipation in the rhombic beam connections. The rhombic structure solely amplifies the input displacement through the rhombic beam to generate the output displacement. The displacement amplification ratio of the rhombic beam under ideal conditions is calculated by using the following equations:

(2) $$\alpha^{\prime }=cos^{-1}\left(cos\alpha -\frac{U_{in}}{L_{r}}\right)$$

(3) $$A=\frac{U_{out}}{U_{in}}=\frac{\cos\alpha -\cos\alpha^{\prime }}{\sin\alpha^{\prime }-\sin\alpha }$$

where $U_{in}$ is the input displacement of the rhombic beam, $U_{out}$ is the output displacement of the rhombic beam, α is the included angle of the rhombic beam without elastic deformation, $\alpha^{\prime }$ is the included angle of the rhombic beam with elastic deformation and $L_{r}$ is the length of the rhombic beam.

**Figure 1:**
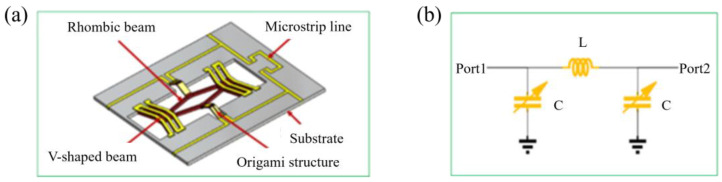
The overall structure diagram and the equivalent circuit of the reconfigurable low-pass filter. (**a**) The overall structure diagram, (**b**) the equivalent circuit.

The origami structure is applied as a reconfigurable component for variable capacitors. The overall structure diagram and equivalent circuit are shown in Figure 1a,b. The filter is fabricated by laser cutting and silk-screen printing. The integration formula for variable capacitors is as follows:

(4) $$C=\int_{0}^{b}\frac{\varepsilon adx}{d+2kx^{2}}=\frac{\varepsilon a}{\sqrt{2kd}}arctan\sqrt{\frac{2k}{d}b}$$

where a is the length of the plate, b is the width of the plate, d is the spacing between the plates, k is the slope during the bending process of the plate and ε is dielectric constant.

The electrothermal actuation generates controlled deformation of the V-shaped beam, which is amplified by the rhombic beam to drive the origami structure. The displacement alters the opposing area and spacing between the polar plates on both sides of the crease, thereby generating a capacitance variation. The origami structure and the inductor constitute a low-pass filter. The inductor of the low-pass filter remains constant, while the capacitance of the origami structure varies with the folding height of the origami structure. The cutoff frequency of the low-pass filter is calculated by

(5) $$f=\frac{1}{\pi \sqrt{LC_{eq}}}$$

(6) $$C_{eq}=2C$$

where L is the inductance of the low-pass filter, $C_{eq}$ is the equivalent capacitance of the low-pass filter and C is the capacitance of the low-pass filter.

Substituting Equation (4) into Equation (5), the cutoff frequency of the low-pass filter is calculated by

(7) $$f=\frac{1}{\pi \sqrt{2L\frac{\varepsilon a}{\sqrt{2kd}}arctan\sqrt{\frac{2k}{d}b}}}$$

### 2.2. Structure Design

**Figure 2:**
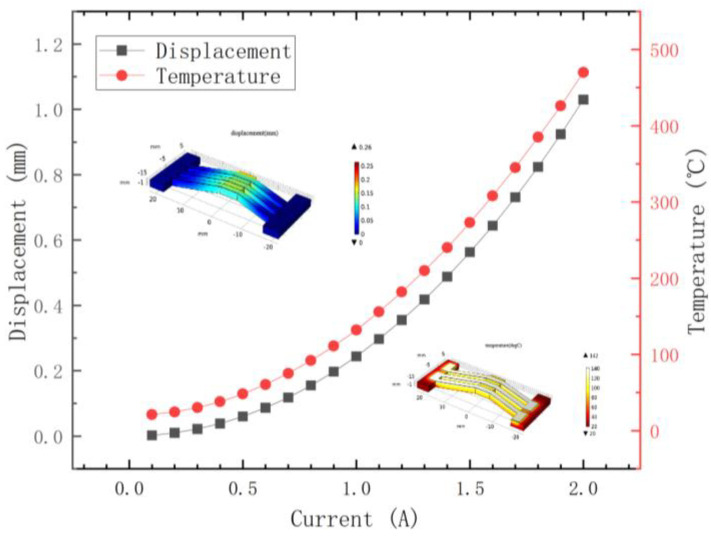
The simulated results of displacement and temperature of the V−shaped beam.

Simulations of the silver-coated polyurethane V-shaped beam were performed using COMSOL Multiphysics 6.2 software. Following the completion of the structural model design for the silver-coated polyurethane V-shaped beam, the relationship among the V-shaped beam’s output displacement, model temperature and input current were obtained through coupled electro-thermal-stress–strain analysis, as shown in Figure 2. The output displacement and temperature of the V-shaped beam demonstrate near-linear increases with the increasing input current, indicating that the model achieves limited displacement under electro-thermal actuation.

**Figure 3:**
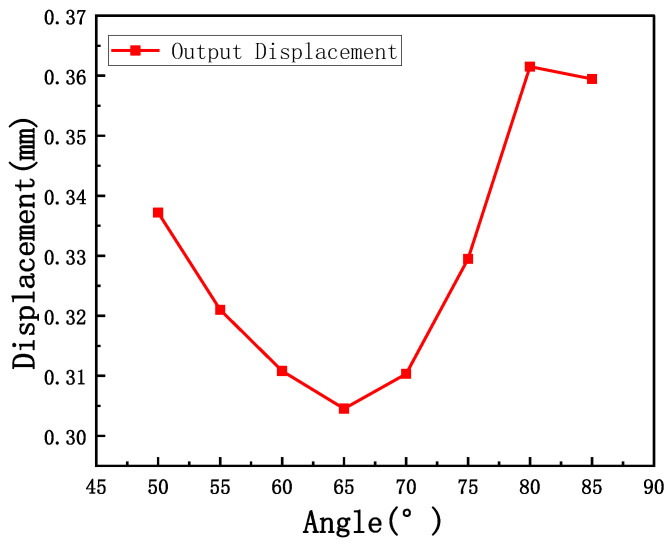
The simulated output displacement of the V-shaped beam versus the angle.

The inclination angle of the V-shaped beam has a significant impact on its output displacement. Parametric analysis of the inclination angle is conducted to determine the optimal configuration. The V-shaped beam achieves maximum output displacement at an inclination angle of 80°, as shown in Figure 3.

**Figure 4:**
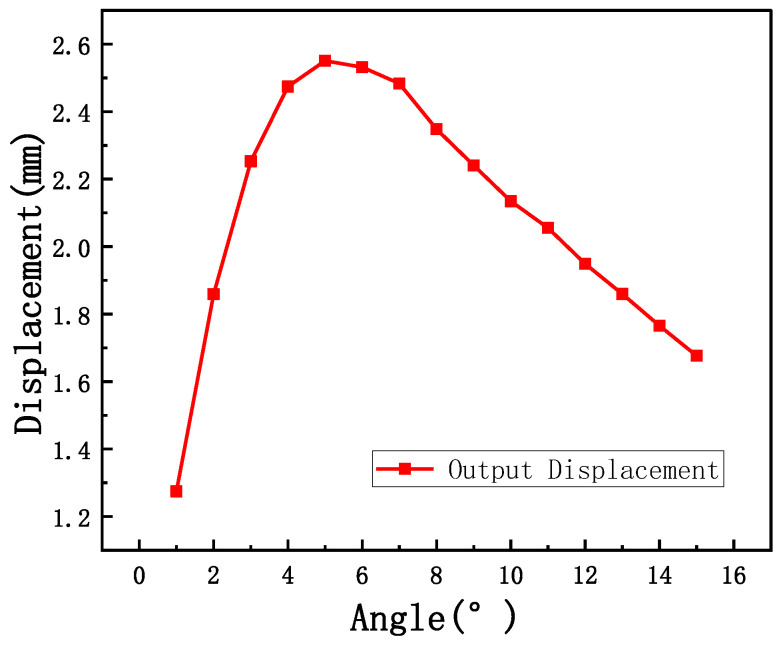
The relationship between the output displacement of the rhombic beam and the inclination angle.

The V-shaped beam exhibits limited output displacement under electro-thermal actuation, necessitating the rhombic beam displacement amplification mechanism. To calculate the displacement amplification of the rhombic beam, a fixed input displacement of 1 mm is applied in the simulation. Consequently, the gain numerically equals the output displacement of the rhombic beam. Figure 4 illustrates the relationship between the output displacement of the rhombic beam and the inclination angle.

Based on the V-shaped beam and the rhombic beam, an origami structure is incorporated into the design. The output displacement from the rhombic beam serves as the input drive for the origami structure. Comprehensive simulations are conducted on the integrated structure.

**Figure 5:**
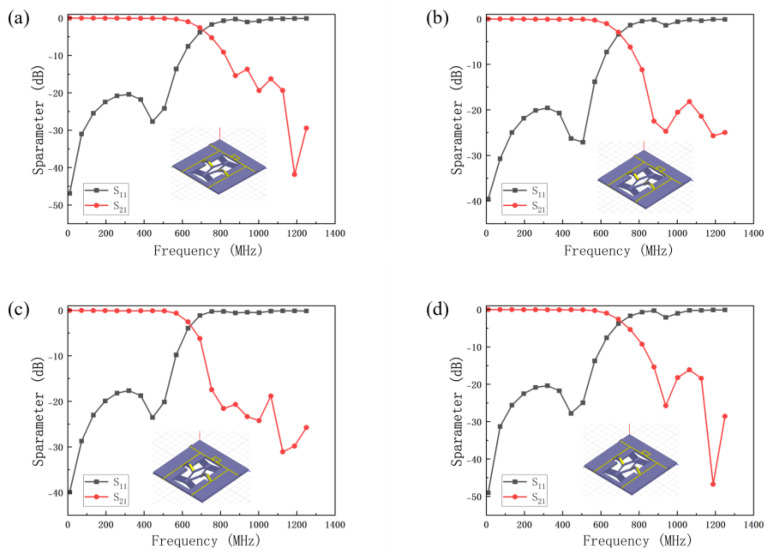
The different configurations of the origami structure model and its S parameters. (**a**) The low−pass filter model and S parameters of the initial state, (**b**) the low−pass filter model and S parameters of 1 mm origami folding height, (**c**) the low−pass filter model and S parameters of 2 mm origami folding height and (**d**) the low−pass filter model and S parameters of 3 mm origami folding height.

A parametric voltage sweep is performed on the metal layer of the V-shaped beam to computationally determine both the displacement amplification of the rhombic beam and the folding height of the origami structure across varying voltages. The V-shaped beam’s output displacement is transmitted through the rhombic beam displacement amplification mechanism to the origami structure, resulting in a vertical displacement at the beam’s center point. Figure 5 presents the different configurations of the origami structure model and its S parameters.

## 3. Results and Discussion

### 3.1. Fabrication

**Figure 6:**
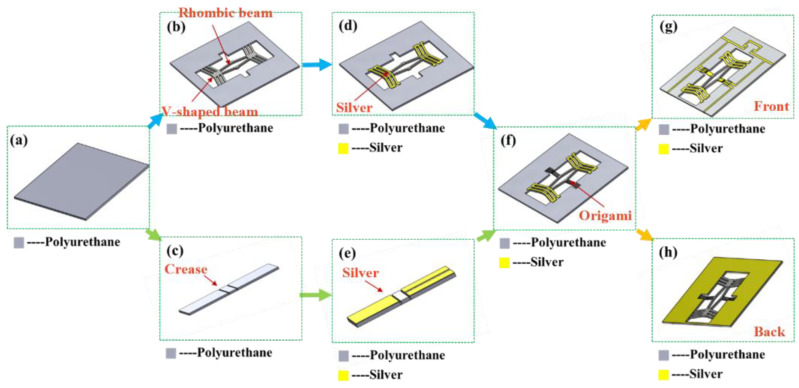
Process flow of the reconfigurable microstrip low−pass filter based on polyurethane inspired by the origami structure. (**a**) A 2 mm thickness polyurethane substrate, (**b**) laser pattern roughing of the V-shaped beam and the rhombic beam, (**c**) laser pattern roughing of the crease, (**d**) laser pattern finishing of the V-shaped beam and the rhombic beam, (**e**) laser pattern finishing of the crease, (**f**) assembled the displacement amplification structure and the origami structure, (**g**) the front screen printing, (**h**) the back screen printing.

The low-pass filter employs polyurethane as the thermal expansion layer material. For monolithic fabrication, both the beam and the origami structure are similarly fabricated using polyurethane. The specific process is shown in Figure 6. Firstly, a polyurethane substrate with a thickness of 2 mm is prepared, as shown in Figure 6a. Then, a designed patterning layout is laser-cut on the polyurethane substrate for rough machining using a computer-controlled laser cutting system, as shown in Figure 6b. Thirdly, precision patterning of the structure is accomplished using a laser marking system, as shown in Figure 6d. After that, the origami structure is fabricated using identical processing techniques, as shown in Figure 6c,e. Next, the origami structure is adhesively bonded to the displacement amplification mechanism for final assembly, as shown in Figure 6f. Finally, a screen-printing stencil is fabricated with designed conductive layer patterns, followed by screen printing of a conductive silver paste to form the metallic conductive layer, as shown in Figure 6g,h.

### 3.2. Characterization

**Figure 7:**
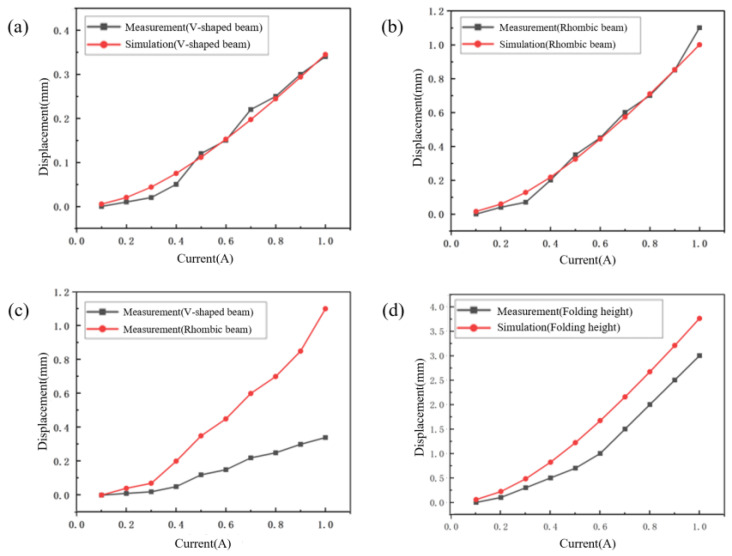
Simulated data and experimental measurements of the cascade beam structure during the origami actuation. (**a**) The measured and simulated displacement outputs of the V-shaped beam under load conditions, (**b**) the measured and simulated displacement outputs of the rhombic beam under load conditions, (**c**) the measured displacement outputs of the V-shaped beam and the rhombic beam under load conditions, (**d**) the measured and simulated folding height of the origami structure.

Figure 7a shows a comparative result of the measured and simulated displacement outputs of the V-shaped beam under load conditions. Figure 7b shows the comparative analysis of measured versus simulated displacement outputs of the rhombic beam under load conditions. Figure 7c shows the relationship between measured displacement outputs of the V-shaped beam and the rhombic beam structures under load conditions. Figure 7d shows the measured and simulated folding height of the origami structure. As evidenced by the data presented in Figure 7, when the input current is below 0.3 A, the structure exhibits negligible deformation. Beyond the threshold current of 0.3 A, both the V-shaped beam’s output displacement and the rhombic beam’s displacement amplification demonstrate close agreement with simulated results under load conditions.

**Figure 8:**
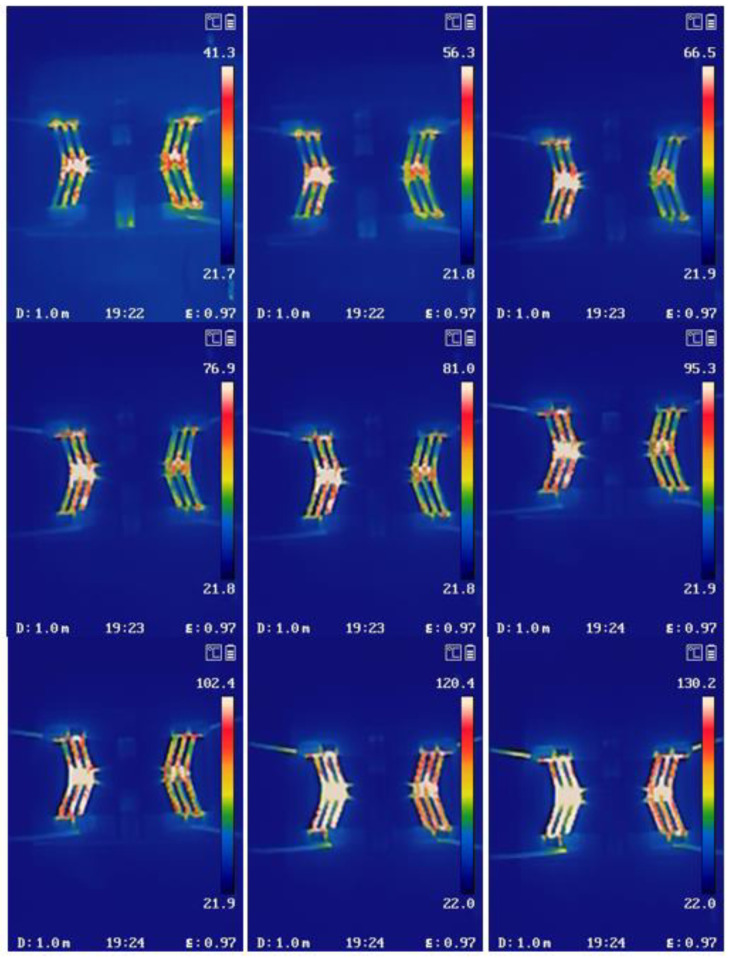
Temperature variation profile on the device surface of the V-shaped and rhombic beam under load conditions.

Figure 8 presents the surface temperature distribution of the V-shaped beam and the rhombic beam under load conditions, with the input current progressively increased from 0.1 A to 1 A. The Joule heating effect induced by current excitation results in a continuous temperature rise from 21.9 °C to 130.2 °C.

**Figure 9:**
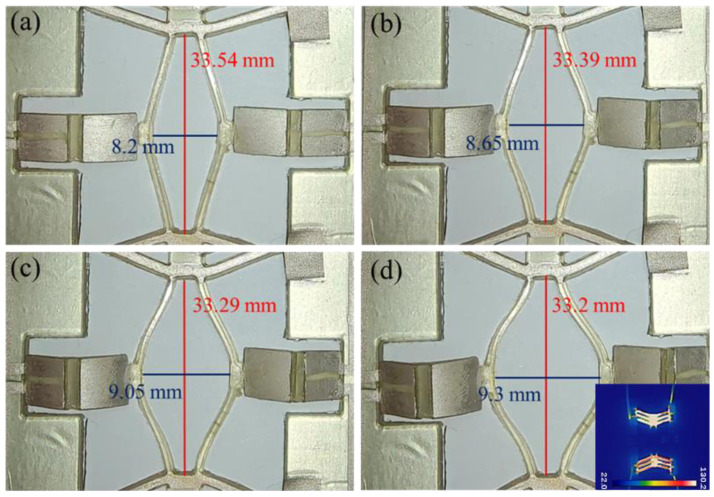
Deformation diagram of V-shaped beam structure, rhombic beam structure and origami structure. (**a**) Initial state, (**b**) driving current of 0.6 A, (**c**) driving current of 0.8 A, (**d**) driving current of 1.0 A and the temperature distribution at current of 1.0 A (the lower right corner of the picture).

The deformation of the V-shaped beam, the rhombic beam and the origami structure is shown in Figure 9. It can be seen that the output displacement of the V-shaped beam increases from 0 to 0.34 mm with the driving current from 0 to 1.0 A. Meanwhile, the output displacement of the rhombic beam structure increases from 0 to 1.1 mm with the displacement amplification of 3.2 and the origami folding height gradually increases from 0 to 3.0 mm. In addition, the temperature distributions of the reconfigurable low-pass filter are measured. As the current passes through the V-shaped beam, the temperature gradually increases from 41.3 °C to 130.2 °C while the current grows from 0.2 A to 1.0 A. Since the melting point of polyurethane is 170 °C, the filter can be reconfigurable in a suitable temperature range.

**Figure 10:**
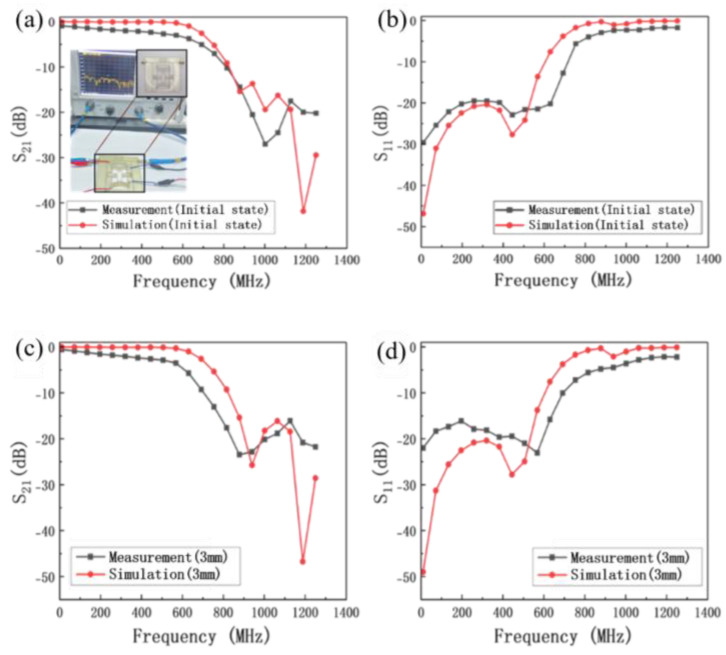
The experimental and simulated results of S parameters of the origami−based reconfigurable low−pass filter. (**a**) Initial state S_21_, (**b**) initial state S_11_, (**c**) the S_21_ of 3 mm origami folding height and (**d**) the S_11_ of 3 mm origami folding height.

To verify the reconfigurable RF device design method based on the origami structure, the reconfigurable low-pass filter with an origami structure is measured by an Agilent N5244A PNA-X Vector Network Analyzer, and the measured results are shown in Figure 10.

The S parameters of the device in the initial state, 1 mm, 2 mm and 3 mm origami folding height are measured. The return loss of the reconfigurable low-pass filter is better than −15.0 dB and the insertion loss decreases from −1.8 dB to −2.5 dB up to 500 MHz when the frequency is tuning from 568 MHz to 524 MHz in Figure 10. The results for 1 mm and 2 mm origami folding height are not shown here. The experimental results are consistent with the simulated results. The error between the experimental and simulated results can be attributed to the difficulty in guaranteeing the machining accuracy in laser cutting and silk-screen printing, resulting in the mismatch between the port and the 50 ohm impedance.

The cutoff frequency of the low-pass filter is below 1 GHz. In the next generation communication system, the sub-6 GHz and millimeter-wave frequency bands are typically used, which necessitates the low-pass filter with higher operating frequency bands and enhanced performance. To achieve higher operating frequency bands in the low-pass filter, smaller capacitance needs to be employed. Compared to origami structure capacitors fabricated using laser cutting and screen-printing techniques, MEMS capacitors based on semiconductor manufacturing processes offer advantages such as a smaller footprint, lower capacitance and superior microwave performance. Utilizing MEMS capacitors enables the low-pass filter to operate at higher frequency bands. Employing different types of MEMS capacitors, such as MEMS interdigital capacitors or MEMS switched capacitors, the capacitance tuning ratio of the reconfigurable filter can be increased, thereby enabling the low-pass filter to cover a wider range of operating frequencies.

**Figure 11:**
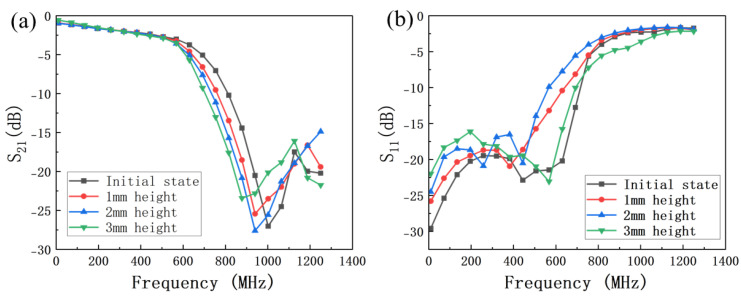
The experimental results of S parameters of the origami−based reconfigurable microstrip low−pass filter with different origami height. (**a**) The experimental results of S_21_, (**b**) the experimental results of S_11_.

The relationship between the microwave performance of the reconfigurable low-pass filter and the origami folding height is shown in Figure 11. As the origami folding height increases from 0 to 3.0 mm, the cutoff frequency of the filter decreases from 568 MHz to 524 MHz, while the return loss remains below −15 dB, the insertion loss better than 2.5 dB up to 500 MHz, maintaining good microwave performance.

## 4. Conclusions

In this paper, a novel reconfigurable RF device design method based on origami is proposed, which provides an innovative structure to design reconfigurable low-pass filters. The reconfigurable low-pass filter with an origami structure is based on polyurethane and the circuit is patterned by silk-screen printing. The experimental results are consistent with the simulated results, which demonstrate the validity of the proposed design method for reconfigurable RF devices.

## Data Availability

The original contributions presented in the study are included in the article, further inquiries can be directed to the corresponding author.
